# Study on Damage Characteristics and Failure Modes of Gypsum Rock under Dynamic Impact Load

**DOI:** 10.3390/ma16103711

**Published:** 2023-05-13

**Authors:** Yongxiang Ge, Gaofeng Ren, Congrui Zhang, Yihu Shi, Luwei Zhang

**Affiliations:** 1 School of Resources and Environmental Engineering, Wuhan University of Technology, Wuhan 430070, China; gyxwhut@163.com (Y.G.); cksyh@whut.edu.cn (Y.S.); zlw1813583@163.com (L.Z.); 2 Key Laboratory of Mineral Resources Processing and Environment of Hubei Province, Wuhan 430070, China

**Keywords:** rock dynamics, split Hopkinson pressure bar, strain rate effect, finite element simulation, failure modes

## Abstract

The objective of this work was to investigate the damage characteristics and failure modes of gypsum rock under dynamic impact loading. Split Hopkinson pressure bar (SHPB) tests were performed under different strain rates. The strain rate effects on the dynamic peak strength, dynamic elastic modulus, energy density, and crushing size of gypsum rock were analyzed. A numerical model of the SHPB was established using the finite element software, ANSYS 19.0, and its reliability was verified by comparing it to laboratory test results. The results showed that the dynamic peak strength and energy consumption density of gypsum rock increased exponentially with strain rate, and the crushing size decreased exponentially with the strain rate, both findings exhibited an obvious correlation. The dynamic elastic modulus was larger than the static elastic modulus, but did not show a significant correlation. Gypsum rock fracture can be divided into crack compaction, crack initiation, crack propagation, and breaking stages, and is dominated by splitting failure. With increasing strain rate, the interaction between cracks is noticeable, and the failure mode changes from splitting to crushing failure. These results provide theoretical support for improvements of the refinement process in gypsum mines.

## 1. Introduction

As typical brittle rock masses, gypsum mines are often produced in the form of thick and large layers with good integrity and excellent stability; therefore, room-and-pillar mining is often used [1]. In the production process of the mine, the multilevel goaf left by in the room-and-pillar mining method exhibits good stability without dynamic disturbance. However, on secondary dynamic disturbances, such as blasting vibration and excavation unloading caused by the production of the adjacent mining area, the pillar and surrounding rock become prone to dynamic damage and deterioration, which, in turn, can induce disasterous accidents in the goaf [2,3,4,5]. To investigate the damage and fracture characteristics of a rock mass under dynamic disturbances, Field et al. [6] detailed the strain rate effect on rock from creep impacting across 16 orders of magnitude in 2004. In general, the mechanical characteristics of rocks under impact and explosion loads are categorized as high strain rate or ultra-high strain rate. The split Hopkinson pressure bar (SHPB) is often used to study the mechanical properties of rock at high strain rates [7,8,9,10,11,12].

Robbiano et al. [13] used a SHPB and a high-speed camera to conduct a thorough investigation into the mechanical behavior and fracture mode of veined rock under dynamic loads and high strain rates. To study the dynamic characteristics and fracture mechanism of gas-bearing coal specimens, Kong et al. [14] conducted a dynamic impact test of gas-bearing coal using SHPB-GAS equipment and analyzed the relationship between the dynamic characteristics of coal rock and impact load. Gong et al. [15] studied the influence of a high strain rate and low confining pressure on the dynamic mechanical properties of sandstone using an SHPB test system. Zhou et al. [16] performed uniaxial dynamic impact tests on nine rocks to investigate the effect of strain rate on the energy conversion and damage deformation of the rocks. Feng et al. [17] showed that the dynamic strength of rock specimens increased obviously with increasing strain rate, whereas the dynamic elastic modulus did not show an obvious effect with changes in loading rate. Wang et al. [18] reached a similar conclusion using the SHPB test. Li et al. [19] reported that the dynamic fracture strength of granite loaded at a medium strain rate is proportional to the cube root of the strain rate. Huang et al. [20] reported that, in a dynamic test, rock was dominated by brittle failure, and the size of the rock fragments decreased with increasing strain rate. The percentage of small-sized (<5 mm) fragments increased, whereas the percentage of large-sized (>20 mm) fragments decreased.

In addition to rock mechanics experiments, numerical simulation methods, such as discrete element method (DEM) simulations and finite element method (FEM) simulations, have been used to study the dynamic mechanical properties and fracture mechanisms of rocks. For example, Li et al. [21] established a numerical SHPB test system based on a particle flow code (PFC) to microscopically reveal the dynamic response of rock specimens using a SHPB with a specifically shaped firing pin. They then performed numerical dynamics simulations at different impact velocities. Luo et al. [22] developed a numerical model of an SHPB using PFC software to analyze the dynamic mechanical properties and damage modes of sandstone in terms of fine fractures and energy. Similarly, Zhu et al. [23,24,25] used the PFC software to investigate the microscopic fracture evolution patterns of rocks under dynamic impact.

Discrete element numerical calculation is mostly used for rock masses with poor continuity in microstructure or unclear constitutive relationship. This method can explain the mechanical properties of rock at the microscopic level. However, the computational process is laborious, and the gaps between particles can affect the accuracy of the results. Therefore, many studies have used FEM to conduct dynamic research. Chen et al. [26] used the FLAC3D software to study the response characteristics of deep clastic rock roadways under static-dynamic load conditions. Li et al. [27] simulated the damage evolution and process of a five-layer combined coal–rock body under impact loading using ANSYS/LS-DYNA. They suggested that radial and circumferential cracks in the rock were mainly formed under low-velocity and high-velocity impact loads, respectively. Peng et al. [28] used ANSYS/LS-DYNA to analyze the effects of diameters and loading rates on the wave dispersion of input rods in rectangular and half-sine-loaded SHPB. Ren et al. [29] used the Holmquist–Johnson–Cook (HJC) and Karagozian and Case concrete (K & C) models in the LS-DYNA software and established the dynamic mechanical properties of ultra-high-performance concrete (UHPC) under two typical dynamic damage models. Wang et al. [30] explored the feasibility and applicability of the modified Riedel–Hiermaier–Thoma (RHT) and HJC models, respectively, in the numerical simulation of granite blasting using LS-DYNA software. The results showed that the RHT model better simulated the kinetic damage characteristics of rocks during cyclic blasting.

As with a typical brittle rock mass, a gypsum mine is more prone to dynamic damage and deterioration when it is subjected to secondary dynamic disturbances such as blasting vibration and excavation unloading. However, there are few research results related to gypsum mines in the field of rock dynamics. Based on the deposit and production characteristics of gypsum ore, the dynamic impact test of gypsum rock was conducted, and the FEM based on the RHT dynamic constitutive model was established. Investigating the dynamic mechanical properties, energy evolution law, and failure mode of anhydrite rock under impact load is important for the safe production of gypsum mines and is the basis for the development of gypsum mine resources.

## 2. Study on Dynamic Impact Test of Gypsum Rock

### 2.1. Specimen Preparation

**Figure 1:**
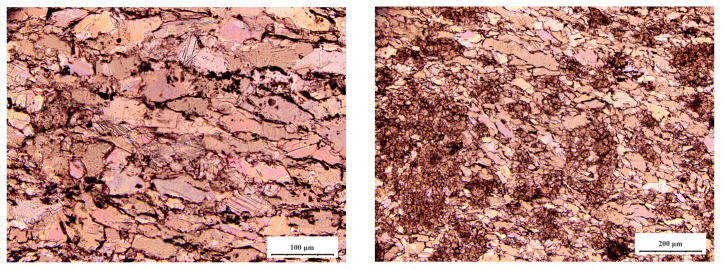
Crystal structure of gypsum rock.

As shown in Figure 1, the specimens were polished and magnified to 100× and 200× under a polarizing microscope. Gypsum rock is a plate-like structure and the structure between the crystals was compact, mixed with dolomite and magnesite minerals. The crystal shape was mostly granular, and the particle size distribution is 0.02–0.1 mm.

**Figure 2:**
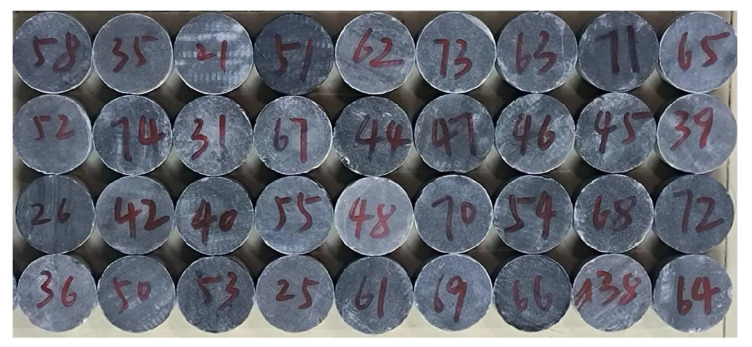
Processed gypsum rock specimens.

As shown in Figure 2, the gypsum rock was machined into cylindrical specimens, 50 mm in diameter and 25 mm in height. The longitudinal wave velocity of all specimens were measured using an acoustic wave tester, and the density was measured using a weighing method. To reduce testing error caused by individual differences among the specimens, longitudinal wave velocity and density were selected to ensure good consistency. The average longitudinal wave velocity of the selected specimens was 6651 m/s, and average density was 2.94 g/cm^3^.

### 2.2. SHPB Test Apparatus and Principle

**Figure 3:**
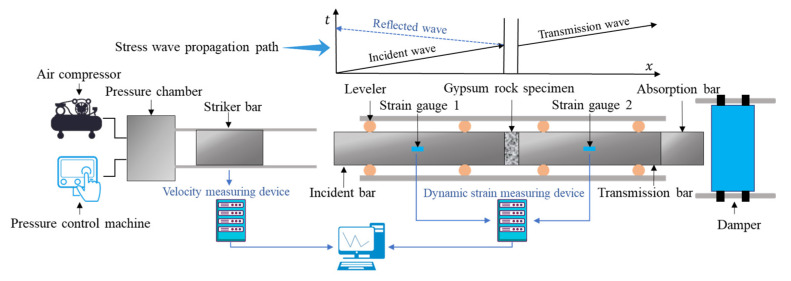
SHPB system schematic diagram.

As shown in Figure 3, the test apparatus consisted of a launcher, stress transfer device, fixed leveling device, and a measuring and recording device. The striker, incident, transmission, and absorption bars were all made of high-strength alloy steels. The density was 7850 g/cm^3^, elastic modulus was 210 GPa, longitudinal wave velocity was 5180 m/s, the incident bar length was 3000 mm, the transmission bar length was 2500 mm, the striker bar length was 400 mm, and the bar diameter was 50 mm. Resistive strain gauges were pasted on the incident bar and the transmission bar. During the test, the strain data of the incident wave, reflected wave, and transmitted wave were measured by a dynamic strain measuring instrument.

In this study, the “three-wave method” was used to calculate the stress $\sigma_{s}$, strain rate ${\dot{\varepsilon }}_{s}$, and strain $\varepsilon_{s}$ of the gypsum rock specimen from the incident wave strain $\varepsilon_{In}(t)$ and reflected wave strain $\varepsilon_{Re}(t)$ measured by strain gauge 1 on the incident bar and the transmitted wave strain $\varepsilon_{Tr}(t)$ measured by strain gauge 2 on the transmitted bar. These were calculated as:

(1) $$\varepsilon_{s}(t)=\frac{C_{e}\int_{0}^{\tau }\left[\varepsilon_{In}\left(t\right)-\varepsilon_{Re}\left(t\right)-\varepsilon_{Tr}(t)\right]dt}{L_{s}},$$

(2) $${\dot{\varepsilon }}_{s}(t)=\frac{C_{e}\left[\varepsilon_{In}\left(t\right)-\varepsilon_{Re}\left(t\right)-\varepsilon_{Tr}(t)\right]}{L_{s}},$$

and

(3) $$\sigma_{s}\left(t\right)=\frac{E_{e}A_{e}\left[\varepsilon_{In}\left(t\right)+\varepsilon_{Re}\left(t\right)+\varepsilon_{Tr}\left(t\right)\right]}{2A_{s}}.$$

where $C_{e}$, $E_{e}$, and $A_{e}$ are the wave velocity, elastic modulus, and cross-sectional area of the elastic bar, respectively. $L_{s}$ and $A_{s}$ are the length and cross-sectional area of the gypsum rock specimen, respectively, and t is the stress wave duration.

According to the principle of functional transformation and the theory of elastic stress wave, the incident wave stress $\sigma_{Ir}(t)$, reflected wave stress $\sigma_{Re}\left(t\right)$, and transmitted wave stress $\sigma_{Tr}(t)$ acquired from the SHPB test can be used to calculate the incident energy $E_{In}$, reflected energy $E_{Re}$ and transmitted energy $E_{Tr}$, which are:

(4) $$E_{In}=\frac{A_{e}}{{\rho_{e}C}_{e}}\int_{0}^{\tau }\sigma_{In}^{2}(t)dt,$$

(5) $$E_{Re}=\frac{A_{e}}{{\rho_{e}C}_{e}}\int_{0}^{\tau }\sigma_{Re}^{2}(t)dt,$$

and

(6) $$E_{Tr}=\frac{A_{e}}{{\rho_{e}C}_{e}}\int_{0}^{\tau }\sigma_{Tr}^{2}(t)dt.$$

In the above formula, ${\rho_{e}C}_{e}$ is the wave impedance of elastic bar.

Considering that the crushing energy consumption of the specimen accounts for a large proportion of the total absorbed energy, the energy lost at the interface between the elastic bar and the specimen, ejection kinetic energy of the broken rock, thermal energy, acoustic energy, and other types of energy consumption can be ignored [31,32,33]. Based on the law of energy conservation, the absorption energy $E_{Ab}$ of the gypsum rock specimen is:

(7) $$E_{Ab}=E_{In}-E_{Re}-E_{Tr}.$$

To reduce the influence of specimen size on energy absorption, the crushing energy density $E_{V}$ is calculated by the following formula:

(8) $$E_{V}=\frac{E_{Ab}}{V_{s}}.$$

In the formula, $V_{s}$ is the volume of the specimen.

### 2.3. Test Results and Discussions

#### 2.3.1. Dynamic Stress Equilibrium

**Figure 4:**
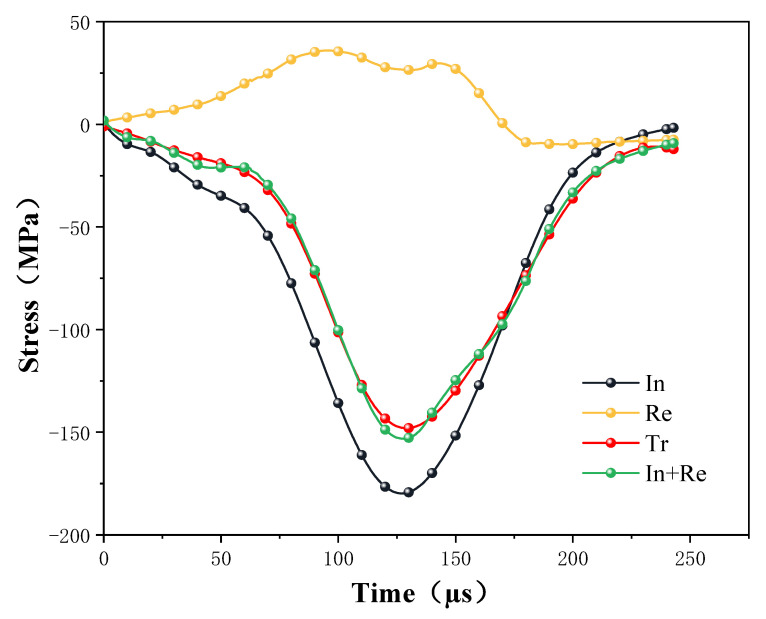
Dynamic stress equilibrium analysis of impact process.

The reliability of the SHPB test is mainly based on the assumptions of a one-dimensional stress wave and stress uniformity. Therefore, before conducting dynamic impact tests, it is necessary to test the stress balance during the loading process to ensure the validity of the test data [34]. The dynamic stress equilibrium state was analyzed using the incident, reflected, and transmitted signals from the dynamic uniaxial compression test process to measure the test equipment reliability. As shown in Figure 4, the curves of stress of incident wave (abbreviated as *In*), transmitted wave (abbreviated as *Tr*), and reflected wave (abbreviated as *Re*) with time are shown. The sum of the incident and reflected strains coincides with the transmission strain, indicating that the specimen was in stress equilibrium during dynamic loading.

#### 2.3.2. Analysis of Test Results

**Figure 5:**
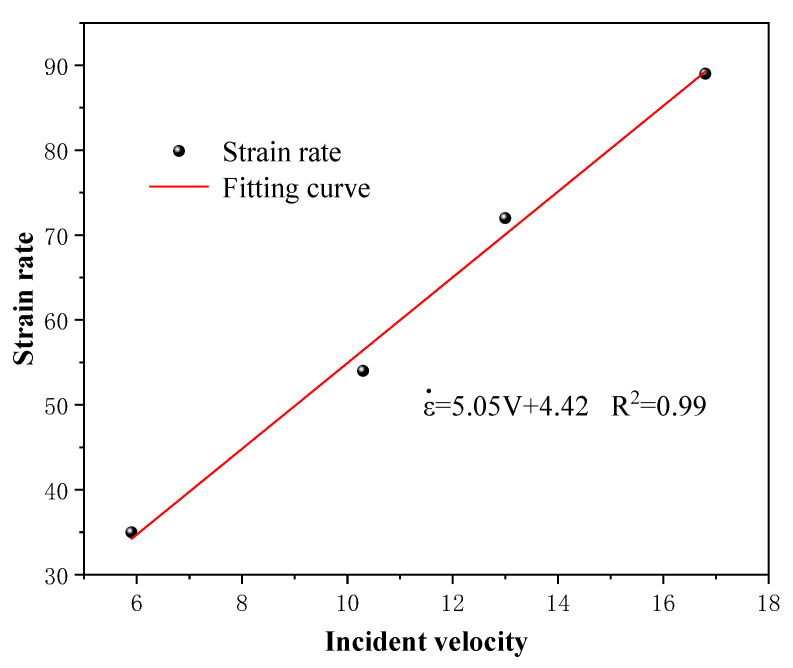
Relationship between strain rate and impact velocity.

When the impact pressure of the test process was set to 0.2 MPa, 0.3 MPa, 0.4 MPa, and 0.5 MPa, the average velocity (abbreviated as *V*) of the striker bar was 5.9 m/s, 10.3 m/s, 13 m/s, and 16.8 m/s, respectively. The average strain rates (abbreviated as $\dot{\varepsilon })$ under different impact pressures were 35 s^−1^,54 s^−1^,72 s^−1^, and 89 s^−1^, respectively. From the test results as shown in Figure 5, it can be concluded that the incident velocity of the striker bar was positively correlated with the strain rate, which increased linearly with incident velocity.

(1) Analysis of stress-strain curve

**Figure 6:**
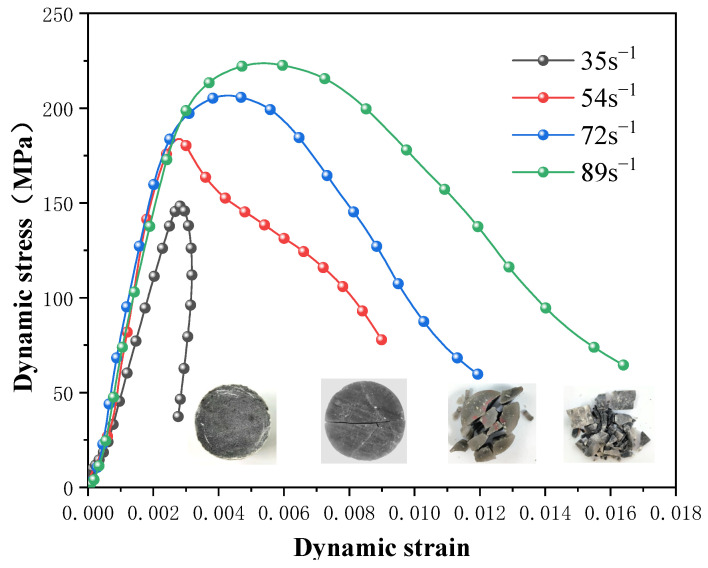
Stress-strain curve and dynamic compressive strength.

Rocks are naturally heterogeneous. Many microcracks were observed in the specimens. In general, in the initial stage of dynamic loading, cracks gradually close under the action of a lower stress, and the nonlinear rising section of the stress-strain curve is a typical rock compaction stage [35]. As the stress value increases to a certain level, the rock enters the elastic deformation stage, and the stress-strain curve shows a certain range of elastic growth. Figure 6 shows the stress-strain curves of the gypsum rock specimens at different strain rates. From the test results, the integrity of gypsum rock was found to be better, with fewer primary visible cracks than in the other rocks; therefore, the compaction stage in the dynamic loading process is not significant.

In the SHPB test, when the dynamic impact stress reached its peak, failure of the specimen occurred in two forms. When the peak stress did not reach the material yield strength, the specimen remained intact. In the post-peak stage, the elastic strain energy stored in the specimen was released, and a rebound phenomenon was evident. The stress-strain curve shows an unloading section and closed curve at a strain rate of 35 s^−1^ in Figure 6. When the peak stress exceeded the yield strength of the material, irreversible failure of the specimen occurred, and the deformation increased cumulatively. The stress-strain curve was open when the strain rate was larger than 54 s^−1^.

From the stress-strain curves at different strain rates, the uniaxial dynamic compressive strength of gypsum rock increases significantly with an increase in the strain rate and shows a significant correlation with the strain rate. The ratio of the dynamic compressive strength to the quasi-static compressive strength (i.e., the peak stress during uniaxial static loading process of rock specimens) was used to characterize the strength-increasing effect of rock, and was defined as the dynamic increase factor (DIF) [36]. The uniaxial compressive strength of the same batch of gypsum rock was 182.11 MPa, previously [37].

The conventional impact test results of gypsum rock as shown in Table 1. The values of the gypsum rock specimens’ dynamic growth factor, DIF, were calculated to be 0.81, 1.01, 1.13, and 1.22 at different strain rates. When the strain rate was 35 s^−1^, the DIF was less than 1. At this strain rate, the constitutive model of gypsum rock mass was closer to the static load constitutive model, and the peak stress of the rock specimen did not reach the yield strength of the material.

The dynamic elastic modulus of gypsum rock was determined by the elastic deformation stage in the stress-strain curve. When the strain rate was greater than 54 s^−1^, the dynamic elastic modulus was approximately 95~99 GPa, which was significantly higher than the elastic modulus under a static load. However, the dynamic elastic modulus remained constant at different strain rates, indicating that the gypsum rock was relatively dense, and its dynamic elastic modulus did not demonstrate a significant strain rate effect, which is consistent with most of the rock laws mentioned in existing research.

(2) Analysis of failure modes

**Figure 7:**
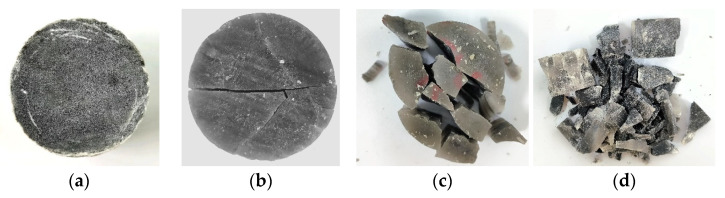
Failure modes of gypsum rock specimen under different strain rates. (**a**) $\dot{\varepsilon }\approx 35\;s^{-1}$ (no destruction); (**b**) $\dot{\varepsilon }\approx 54\;s^{-1}$ (splitting failure); (**c**) $\dot{\varepsilon }\approx 72\;s^{-1}$ (splitting failure); (**d**) $\dot{\varepsilon }\approx 89\;s^{-1}$ (mixed failure mode of columnar splitting and crushing).

As shown in Figure 7. From the failure modes of gypsum rock specimens under different strain rates, when the strain rate was 35 s^−1^, a small number of micro-cracks were generated on the surface of the specimen, but the overall integrity was good. When the strain rate reached 54 s^−1^, the gypsum rock specimen started to crack in a direction parallel to the compressive stress and macroscopic cracks were produced. The specimen exhibited an obvious columnar splitting failure. At a strain rate of 72 s^−1^, the specimen still exhibited columnar splitting failure; however, the number of fracture surfaces was significantly higher than that at a strain rate of 54 s^−1^. When the strain rate was 89 s^−1^, the activation degree of micro-cracks in the specimen was greater, and the specimen showed a mixed failure mode of columnar splitting and crushing. In general, with an increase in the strain rate, the degree of macroscopic fracture of the specimen increased gradually, and the fracture size of the specimen decreased, showing a significant correlation with strain rate.

(3) Analysis of energy dissipation

**Figure 8:**
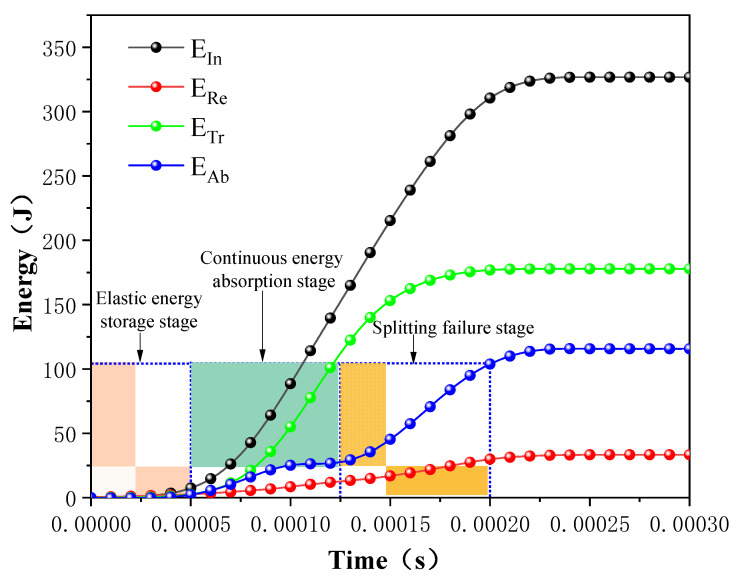
The energy evolution curve at strain rate of 72 s^−1^.

The energy dissipation of rocks is the first driving force of rock material failure in dynamic crushing. Rock produces irreversible energy dissipation under impact loads, and the size, quantity, and scale distribution of rock fragments after rock failure are macroscopic manifestations of energy dissipation [38]. As shown in Figure 8, under an impact load, when the impact stress propagates to the rock interface through the incident bar, part of the energy is reflected to the incident bar in the form of a reflected tensile wave, and the other part of the energy propagates in the form of a transmitted compressive wave. Figure 8 shows the curves of incident energy (abbreviated as $E_{In}$), reflected energy (abbreviated as $E_{Re}$), transmitted energy (abbreviated as $E_{Tr}$), and absorbed energy (abbreviated as $E_{Ab}$) changing with time.

When the stress propagates to the rock specimen, part of the energy is absorbed by the rock and stored in the form of elastic energy. As shown in Figure 8, the rock specimen is in the elastic deformation stage during the time period of 0~50 μs. During 50~125 μs, the intensity of the incident stress wave is greater than the ultimate compressive strength of the rock specimen, and the cumulative damage is generated inside the specimen, resulting in numerous new micro-cracks. The absorption energy growth rate increases significantly, and the absorption energy increases approximately linearly with time. In the stage of 125~200 μs, the growth rate of absorption energy increases again, and the cracks inside the rock specimen expand and penetrate rapidly, resulting in axial splitting failure.

(4) Analysis of strain rate effect on dynamic parameters

**Figure 9:**
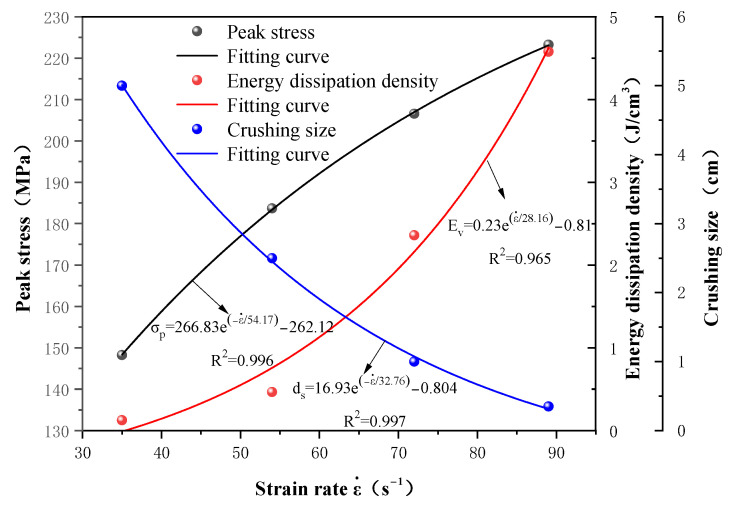
The relationship between dynamic peak strength, energy dissipation density, crushing size, and strain rate.

The relationship between the peak stress (abbreviated as $\sigma_{p}$), energy dissipation density (abbreviated as $E_{v}$), average crushing size (abbreviated as $d_{s}$) of the gypsum rock specimens, and strain rate (abbreviated as $\dot{\varepsilon }$) is shown in Figure 9. Under a high strain rate dynamic impact, before the formation of the main penetrating cracks, these new cracks cannot expand or further penetrate owing to the limited energy release area and crack propagation speed, hence, they dissipate a large amount of energy, and indirectly improve the macroscopic failure strength of the rock specimens. The energy dissipation density of rock increases with the increase of strain rate, and the growth rate also increases gradually. When the strain rate is low, a small number of microcracks are generated inside the rock, and energy consumption is low. When the strain rate increases, the number of cracks inside the rock increases sharply and begins to expand, resulting in a rapid increase in energy consumption. As an increasing strain rate leads to rapid growth and expansion of the number of cracks in the rock, the fracture size of the specimen decreases, and the average size decreases exponentially.

**Figure 10:**
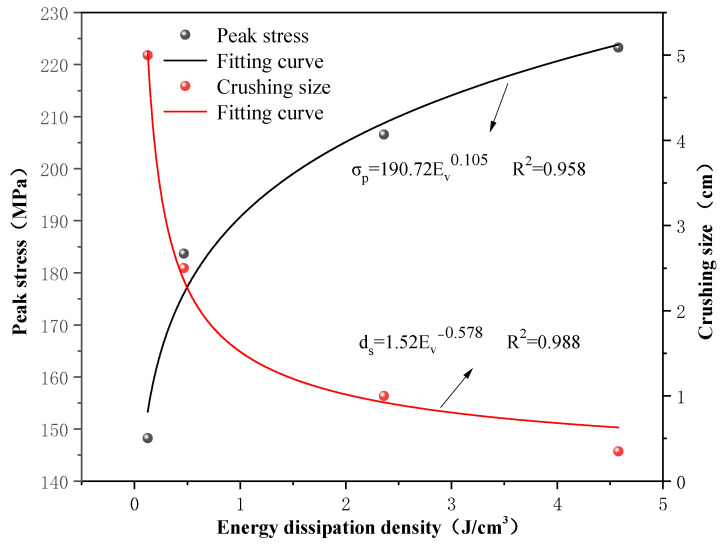
The relationship between dynamic peak strength, crushing size and energy dissipation density.

As shown in Figure 10, the dynamic strength of gypsum rock specimens increases exponentially with energy dissipation density. Similarly, the relationship between the energy dissipation density and the specimen crushing size at different strain rates also satisfy the power-exponential function. Therefore, a higher strain rate leads to higher absorption energy of the specimen, and the number of microcracks generated by excitation is greater, which leads to complete breakage of the specimen.

## 3. Numerical Simulation of Dynamic Impact of Gypsum Rock Based on RHT Model

The strain rate loading range of the SHPB test device is 10~10^3^ s^−1^; hence, studying the dynamic characteristics of gypsum rock under ultra-high strain rate is impossible. At the same time, the SHPB test has defects such as low repeatability, high costs, and an unmeasurable dynamic damage process [21,39,40]. Therefore, establishing a numerical calculation model based on laboratory tests is necessary to explore the dynamic characteristics of gypsum rock comprehensively and accurately.

### 3.1. Establishment of Numerical Calculation Model

**Figure 11:**
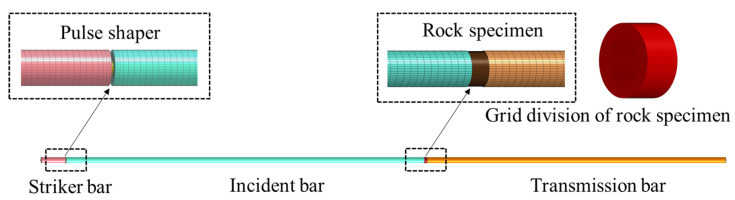
Numerical calculation model of SHPB.

The finite element analysis software ANSYS/LS-DYNA was used to simulate the SHPB test, and the RHT damage constitutive model was selected. The basic unit of the model is cm-g-μs in the ANSYS/LS-DYNA finite element program. A 3D-SOLID full model was established based on the actual size and material properties of the elastic steel bar and anhydrite specimen. The details of the model establishment and grid division of the model are described here. The incident bar model was 300 cm in length and 5 cm in diameter, which was divided into 300 parts in the axial direction and 24 parts in the radial division. The transmission bar model was 250 cm in length and 5 cm in diameter, which was divided into 250 parts in the axial direction and 24 parts in the radial division. The gypsum specimen model was 2.5 cm in height and 5 cm in diameter, which was divided into 30 parts in the axial direction and 60 parts in the radial division. A total of 318,600 units and 337,855 nodes are divided by a hexahedral mesh model. The established physical model is shown in Figure 11.

#### 3.1.1. Determination of RHT Model Parameters

The RHT constitutive model can simulate the processes of projectile penetration and explosion impacts on brittle materials. The RHT model contained 34 parameters. Based on the functional relationships between the parameters, they are divided into main, subordinate, and fixed parameters. According to their physical meaning and function in the RHT model, the acquisition of the primary parameter values must be determined by different tests.

(1) Parameter determination of p−α state equation

Rock contains original defects, such as pores, holes, and microcracks of different sizes. Therefore, the mechanical behavior of such materials under pressure is nonlinear, and the p−α state equation proposed by Herrman in 1969 is used for descriptive purposes [41].

The material density, $\rho_{0}$, and initial porosity, $\alpha_{0}$, in the p−α equation of state were directly measured by balance and mass methods. The equation parameters of the p−α state equation in the compressed state include $A_{1}$, $A_{2}$, $A_{3}$, $B_{0}$ and $B_{1}$. The equation parameters in the tensile state include $T_{1}$ and $T_{2}$. To derive and solve the above parameters, the Rankine–Hugoniot equation and Mie–Gruneisen state equations were used:

(9) $$A_{1}=\rho_{0}c_{0}^{2}=K_{m}=134.4\;GPa,$$

(10) $$A_{2}=K_{m}\left(2s-1\right)=225.8\;GPa,$$

and

(11) $$A_{3}=K_{m}\left(3s^{2}-4s+1\right)=138\;GPa.$$

The empirical parameter s is the same as that of granite (1.34.)

Mie–Gruneisen state equation was used:

(12) $$B_{1}=B_{0}=\gamma_{0}=2s-1=1.68.$$

The parameters $T_{1}$ and $T_{2}$ in the tensile state were calculated using the following formulas:

(13) $$T_{1}{=A}_{1}=\rho_{0}c_{0}^{2},$$

and

(14) $$T_{2}=0.$$

The pressure $P_{el}$ needed at the beginning of pore crushing is calculated as:

(15) $$P_{el}=f_{c}/3,$$

where $f_{c}$ is the uniaxial compressive strength (MPa).

In the p−α state equation, porosity index N and compaction pressure $P_{comp}$ are selected as empirical values.

(2) Parameter determination of RHT constitutive equation

The compressive strain rate index $\beta_{c}$ and the tensile strain rate index $\beta_{t}$ can be calculated according to the following formulas:

(16) $$\beta_{c}=\frac{4}{20+3f_{c}}$$

and

(17) $$\beta_{t}=\frac{2}{20+f_{c}}.$$

From the rock mechanics test results of gypsum rock performed by the research group in the early stage, the uniaxial compressive strength of gypsum rock was 182.11 MPa, tensile strength was 22.2 MPa, elastic modulus was 85.67 GPa, and Poisson’s ratio was 0.168 [37]. The shear modulus of gypsum rock can be obtained according to the elastic wave theory:

(18) $$G=\frac{E}{2(1+v)},$$

where G is the shear modulus, E is the elastic modulus, and v is Poisson ’s ratio.

Accordingly, the uniaxial compressive strength, $f_{c}$, the tensile-compressive strength ratio, $f_{t}^{*}$, and the shear modulus, G, of the basic mechanical parameters of the RHT model were obtained.

The RHT principal equations also include the shear-to-compression strength ratio, $f_{s}^{*}$, failure surface parameter, A, failure surface index, n, compression yield surface parameter, $g_{c}^{*}$, tension-compression meridian ratio parameter, $Q_{0}$, shear modulus reduction coefficient, ξ, initial damage parameter, $D_{1}$, minimum failure strain, $\varepsilon_{p}^{m}$, residual stress intensity parameter, $A_{f}$, and residual stress intensity index, $n_{f}$. Owing to the complexity of the acquisition method, an empirical value was selected based on the research conclusions of Riderde [42].

#### 3.1.2. Parameter Selection of RHT Model

Based on the experimental and theoretical calculation parameters, the RHT model can accurately describe the dynamic response characteristics of gypsum rock, largely depending on the values of empirical parameters. Therefore, through an orthogonal experimental design combined with a comparison between the numerical calculation and experimental results, a set of empirical parameters suitable for simulation of the gypsum mine impact test was determined. Through sufficient error tests, the microscopic parameters were calibrated to match the macroscopic mechanical responses. Table 2 presents the selected results for the 34 parameters used in the RHT model.

### 3.2. Analysis of Numerical Results

#### 3.2.1. Comparative Analysis of Dynamic Characteristics and Failure Modes

**Figure 12:**
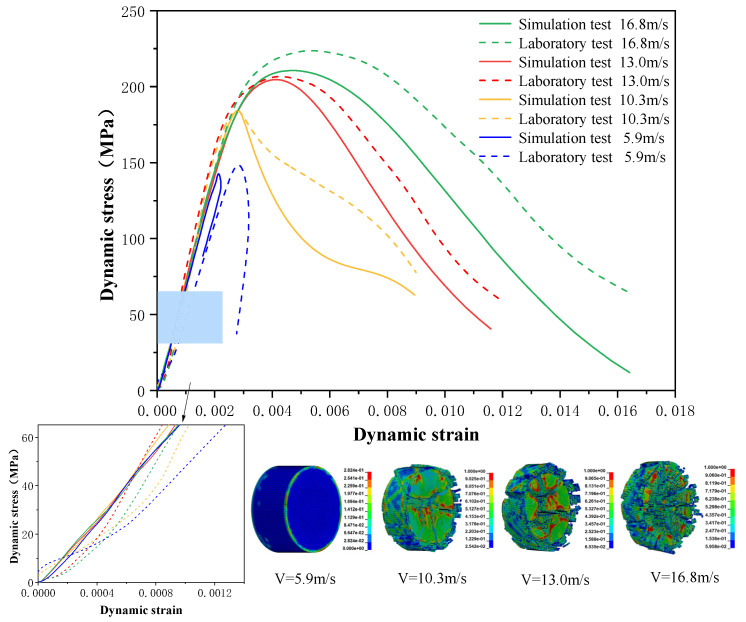
Comparison of stress-strain curve between laboratory test and numerical calculation.

Using the optimized RHT model, the dynamic test simulation of gypsum rock specimen under different impact velocities was performed. The impact velocity of the incident end was set to 5.9 m/s, 10.3 m/s, 13.0 m/s, and 16.8 m/s. Figure 12 shows the stress-strain curves and final rupture morphology of the numerical calculations and laboratory test results. According to the numerical calculations, it can be see that the peak stress does not reach the yield strength of the rock specimen when the impact velocity is small, i.e., under low strain rate impact, the specimen undergoes rebound unloading phenomenon. In such a case the stress-strain curve is closed, and the specimen does not produce macrocracks. This is consistent with the laboratory test results. With increasing strain rate, the specimens were damaged to varying degrees; thus, the stress-strain curves were open. In the laboratory test, the integrity of the gypsum rock was found to be better than in other tested rocks, and there were fewer primary cracks. Therefore, the compaction stage in the dynamic loading process is not obvious, but it performs well. However, the numerical model fails to fully consider the initial conditions of the primary microcracks of the gypsum rock specimens. Therefore, the stress-strain curve acquired by the numerical calculation is inconsistent with the laboratory test, which does not reflect the nonlinear change in the crack compaction process, as shown in the enlarged area of Figure 12.

Previous laboratory test results have shown that the strain rate is proportional to the impact velocity of the incident bar. According to the numerical calculation results, the peak strength of gypsum rock specimens gradually increases with the increase of strain rate, showing a significant rate correlation. This is consistent with the pattern exhibited in laboratory tests. As shown in the Figure 12, the stress-strain curves acquired by the numerical calculation and laboratory test have good consistency, and the fracture morphology of the rock specimen after reaching the peak strength is also basically the same. The elastic deformation ranges of different strain rates are approximately the same, and undergo no obvious change with strain rate. This phenomenon is in agreement with the laboratory test results. Therefore, the SHPB finite element numerical calculation model validity based on the optimized RHT model was verified.

#### 3.2.2. Analysis of Rock Failure Process

The failure process of gypsum rock with different impact velocities was divided into four stages, as listed in Table 3. (1) Fracture Compaction stage is the initial stage of dynamic loading, when the primary microcracks in the specimen gradually closed under a lower stress, showing a short-term crack compaction effect; however, no macrocracks were formed at this stage. (2) Crack Initiation stage, occured with a further increase in stress, subsequent crack propagation occurred and more microcracks were randomly activated. Cracks initiated near the bar end of the specimen and rapidly expanded along the axial direction. (3) Crack Propagation stage is when, under continuous loading of the stress, the primary and secondary cracks in the rock specimen continued to develop and penetrate. Multiple macrocracks are formed along the direction of compressive stress. (4) Crushing stage is when the macroscopic cracks expanded further and the rock specimens underwent axial splitting failure. At a high strain rate, the interaction between cracks is more noticeable. The existence of adjacent cracks inhibits further expansion of other cracks and leads to a change in the direction of crack propagation; thus, the specimen undergoes crushing failure.

There are three main ultimate failure modes of gypsum rock under different velocity impacts. For loading in the elastic category of the specimen, the main performance factor was the generation of internal microcracks. No macroscopic cracks or deformations were generated. At higher strain rates, the specimens exhibited varying degrees of damage, which was primarily dominated by splitting failure parallel to the loading direction. Therefore, the macroscopic cracks formed and developed independently, and there was no interaction between the cracks. With an increase in the strain rate, more microcracks were initiated, the interaction of the macrocracks after continuous expansion was enhanced, and obvious crack crossing occurred. Therefore, the specimens exhibited axial and circumferential block failures.

## 4. Conclusions

A gypsum mine is more prone to dynamic damage and deterioration when it is subjected to secondary dynamic disturbances such as blasting vibration and excavation unloading. However, there is little research related to gypsum mines in the field of rock dynamics. In this paper, the dynamic properties and fracture modes of gypsum rock with varying strain rates were investigated using the SHPB impact test. Considering the loading strain rate limit of the SHPB test and the inability to record the dynamic process of fracture generation in gypsum rock, a dynamic impact numerical simulation of gypsum rock specimens under different impact velocities was performed. By comparing the results with laboratory test results, the validity of the model was established in the research process. The conclusions are shown below.

(1) The impact test results showed that the peak stress, energy dissipation energy density, and crushing size of gypsum rock exhibit obvious correlations. With the increase of strain rate, peak strength and energy dissipation energy density increased exponentially. The crushing size had a negative exponential relationship with the strain rate and tended to be gentle with an increase in the strain rate. The dynamic elastic modulus was larger than the static elastic modulus. However, no strain-rate effect was exhibited.
(2) The energy dissipation analysis during the test process indicated that the dynamic strength of the gypsum rock specimens increased exponentially with the energy dissipation density of crushing. Similarly, the relationship between energy dissipation density and specimen crushing size at varying strain rates satisfied the power-exponential function. This shows that a higher strain rate leads to a higher absorption energy of the specimen, and the number of microcracks generated by excitation is greater, which leads to complete breakage.
(3) The laboratory test results proved that the optimized numerical calculation model could effectively describe the dynamic characteristics of gypsum rock. In subsequent research, it can be used as a supplementary method to study the dynamic and damage characteristics of gypsum rock at higher strain rates.
(4) Under the impact of a low strain rate, if the loading strength was within the elastic range of the specimen, it is manifested as the generation of internal microcracks, without macroscopic cracks or deformations. Under the impact of a high strain rate, the fracture of gypsum rock can be divided into the crack compaction, crack initiation, crack propagation, and breaking stages, and is dominated by splitting failure. With increasing strain rate, the failure mode changes from splitting to crushing failure, and the interaction between cracks cannot be ignored.

## Figures and Tables

**Table 1:** Conventional impact test results of gypsum rock.

Strain Rate (s^−1^)	Dynamic Peak Stress (MPa)	DIF	Dynamic Elastic Modulus (GPa)	Density of Energy Dissipation (J/cm^3^)	Crushing Size (cm)
35	147.51	0.81	70.55	0.125	5
54	183.93	1.01	95.64	0.466	2.5
72	205.78	1.13	99.82	2.36	1
89	222.17	1.22	96.39	4.578	0.36

**Table 2:** The selection results of RHT model parameters.

Parameter Symbolic	Value	Parameter Symbolic	Value	Parameter Symbolic	Value
$\rho_{0}$	2.917 g/cm^3^	$f_{c}$	182 MPa	$\alpha_{0}$	1.02
$P_{el}$	60.7 MPa	$\beta_{t}$	0.0099	$\beta_{c}$	0.0071
$A_{1}$	134 GPa	$A_{2}$	226 GPa	$A_{3}$	138 GPa
$B_{0}$	1.68	$B_{1}$	1.68	$T_{1}$	1.34
$T_{2}$	0	G	36.74 GPa	$P_{comp}$	0.8
${\dot{\varepsilon }}_{0}^{c}$	3 × 10^−8^ ms^−1^	${\dot{\varepsilon }}_{0}^{t}$	3 × 10^−9^ ms^−1^	${\dot{\varepsilon }}_{c}$	3 × 10^22^ ms^−1^
${\dot{\varepsilon }}^{t}$	3 × 10^22^ ms^−1^	$D_{2}$	1	B	0.0105
n	0.57	$g_{t}^{*}$	0.7	A	2.44
$f_{s}^{*}$	0.45	$f_{t}^{*}$	0.13	$Q_{0}$	0.6805
$g_{c}^{*}$	0.95	ξ	0.60	$n_{f}$	0.81
$D_{1}$	0.048	$\varepsilon_{p}^{m}$	0.015	$A_{f}$	0.30
N	3				

**Table 3:** Rock failure process under dynamic loading.

Impact Velocity	Compaction Stage	Crack Initiation	Crack Propagation	Splitting Failure	Splitting Failure (Laboratory Test)
5.9 m/s	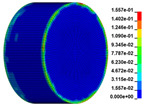	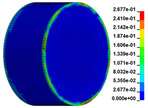	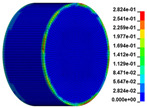	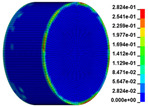	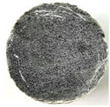
10.3 m/s	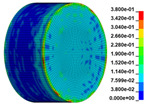	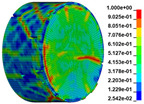	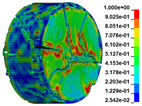	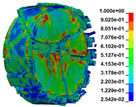	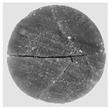
13.0 m/s	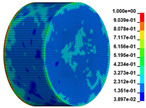	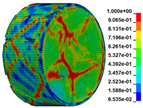	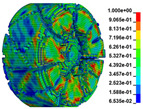	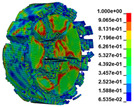	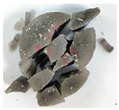
16.8 m/s	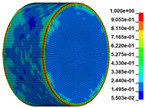	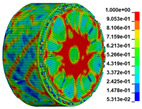	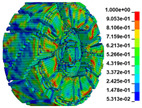	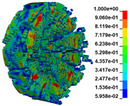	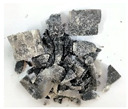

## Data Availability

Data sharing not applicable.
